# Cost-effectiveness of antiviral prophylaxis during pregnancy for the prevention of perinatal hepatitis B infection in South Korea

**DOI:** 10.1186/s12962-018-0088-9

**Published:** 2018-02-15

**Authors:** Donghoon Lee, Hyun-Young Shin, Sang Min Park

**Affiliations:** 1 Department of Preventive Medicine, Kyung Hee University College of Medicine, Seoul, South Korea; 2 Department of Family Medicine, Myongji Hospital, Goyang, South Korea; 3 Department of Biomedical Sciences, Seoul National University College of Medicine, Seoul, South Korea; 4 Department of Family Medicine, Seoul National University College of Medicine, Seoul, South Korea

**Keywords:** Hepatitis B, Perinatal infection, Antiviral prophylaxis, Cost-effectiveness analysis

## Abstract

**Background:**

In Korea, hepatitis B virus (HBV) infection accounts for approximately 65–75% of HBV-related diseases, such as chronic hepatitis and liver cancer, and mother-to-child transmission is presumed to be a major source of the infection. To tackle this issue, the Korean government launched the national Perinatal Hepatitis B Prevention Program (PHBPP) in 2002. This study analyzed the cost-effectiveness of the PHBPP with antiviral prophylaxis compared with the current PHBPP and/or universal vaccination, as well as identified the optimal strategy to eliminate mother-to-child transmission of HBV in Korea.

**Methods:**

A decision tree model with the Markov process was developed and simulated over the lifetime of a birth cohort in Korea during the year 2014. The current PHBPP providing HBV vaccine and hepatitis B immune globulin to neonates born to HBV positive mothers was compared against two other strategies, universal vaccination of HBV and PHBPP with antiviral prophylaxis, with respect to their costs and health outcomes. The Korean National Health Insurance database was investigated to estimate the costs of HBV-related diseases and utilization of health resources. Costs were assessed from the health care system perspective and converted to 2014 US dollars. Health outcome measures were quality-adjusted life years (QALYs) and number of HBV-related diseases and deaths. Both costs and QALYs were discounted at 5%, following the recommendation of the Health Insurance Review & Assessment Service in Korea. The incremental cost-effectiveness ratio (ICER) obtained from the analysis was evaluated using the willingness-to-pay (WTP) in the Korean society.

**Results:**

PHBPP with antiviral prophylaxis in Korea was cost-effective compared with the current PHBPP. An introduction of antiviral prophylaxis to pregnant women with a high viral load of HBV averted 13 HBV-related deaths per 100,000 people and saved 82 QALYs in total (ICER: $16,159/QALY).

**Conclusions:**

Considering that WTP in Korea is $29,000, PHBPP with antiviral prophylaxis appears to be a cost-effective strategy. To further decrease the burden of perinatal hepatitis B in Korea, adding antiviral prophylaxis to PHBPP is recommended.

## Background

Hepatitis B virus (HBV) is a well-known risk factor for liver diseases, including chronic hepatitis, cirrhosis, and Hepatocellular Carcinoma (HCC) [1]. In Korea, HBV infection accounts for approximately 65–75% of these disease incidences; it is recognized as an endemic disease of the country [2–4]. To cope with this, the Korean government has begun to focus on the prevention of HBV infection by vaccinating newborns.

The prevalence of HBV infection in Korea has declined over the past several decades. The peak positive rate of hepatitis B surface antigen (HBsAg) in the early 1980s was recorded to be 8–9% for males and 5–6% for females [5]. Since then, with the development of HBV vaccination by a domestic pharmaceutical company in 1983, the prevalence of HBsAg in the Korean population has been decreasing steadily, and the addition of HBV vaccination into the National Immunization Program for Children in 1995 further reduced the HBsAg prevalence in the population, especially those under the age of 20 years [2, 6], dropping the prevalence by one-third, from 3.9% (1996) to 1.3% (1999) within the first 5 years since the inception of the program [7].

Currently, mother-to-child transmission is presumed to be a major source of HBV infection in Korea [8]. In 2002, the Korea Centers for Disease Control and Prevention (KCDC) launched the national Perinatal Hepatitis B Prevention Program (PHBPP) to tackle the issue of mother-to-child transmission of HBV. This program consists of an administration of hepatitis B immune globulin (HBIG) and HBV vaccine at birth, followed by two additional doses of HBV vaccine at 1 month and at 6 months after birth [9]. High rates of program participation (approximately 96%) and antibody formation among participants (approximately 97%) brought a dramatic reduction, up to 3% in vertical transmission of HBV [10]. However, considering that the timely administration of HBV vaccine and HBIG do not fully prevent the occurrence of in utero infection of HBV, neonates born to mothers with a high viral load of HBV are still susceptible to the infection [11]. Thus, to eliminate the risk of in utero infection of HBV, an inclusion of antiviral prophylaxis in the current PHBPP is recommended to pregnant women with a high viral load of HBV [12].

The purpose of this study was to assess the cost-effectiveness of PHBPP with antiviral prophylaxis compared with the current PHBPP and/or universal vaccination that provides only HBV vaccination within the first 24 h of birth. Then, we identified the optimal strategy for eliminating mother-to-child transmission of HBV in Korea.

## Methods

### Three intervention strategies

**Fig. 1:**
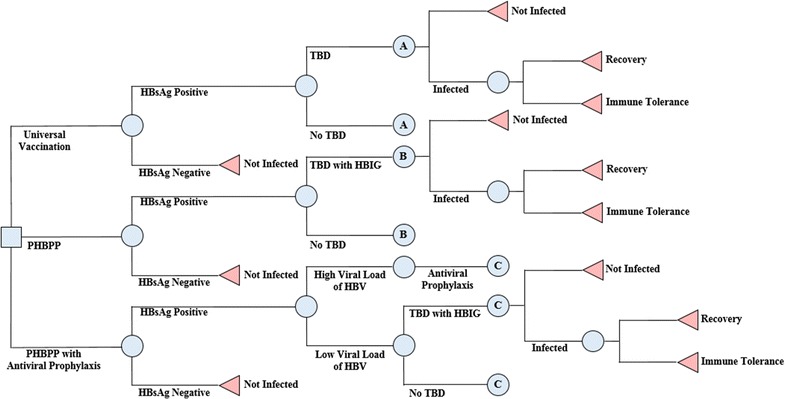
Decision analytic model. PHBPP, Perinatal Hepatitis B Prevention Program; HBsAg, Hepatitis B Surface Antigen; TBD, Timely Birth Dose; HBV, Hepatitis B Virus; HBIG, Hepatitis B Immune Globulin

Three preventive strategies against perinatal HBV infection were illustrated in our decision tree model (Fig. 1). The details of each strategy are as follows.

1. Universal vaccination: regardless of the HBsAg status of mothers, newborns receive the initial dose of HBV vaccine within the 24 h of birth. Those administered with the initial dose of the vaccination comply with the ordinary schedule of HBV vaccination in Korea, which is to receive a vaccination at 0, 1, and 6 months [13, 14].
2. Current PHBPP: through antenatal screening, HBsAg-positive mothers become subjects to receive PHBPP. The post-exposure prophylaxis of HBV, a combination of HBIG and HBV vaccine, is provided to their newborns right after birth. Infants completing every schedule of HBV vaccination are eligible to undergo antigen and antibody tests, which are paid for by the government until they acquire the necessary antibodies to HBV [9].
3. PHBPP with antiviral prophylaxis: after antenatal screening, an additional HBV-DNA testing is performed on HBsAg-positive mothers to measure their viral load of HBV. Mothers with a high viral load (≥ 10^6^ copies/mL) are eligible to receive antiviral prophylaxis for 4 months, beginning at the 3rd trimester of pregnancy up until 1 month postpartum. Subsequent measures, which is equivalent to the current PHBPP, are administered to newborns to prevent perinatal HBV infection after birth [15].

### Model

The decision analytic model with Markov process was constructed to evaluate HBV-related costs and health outcomes, as well as quality-adjusted life years (QALYs) over the lifetime of the birth cohort in Korea during the year 2014 [16]. Each node of the model reflects epidemiological circumstances of HBV in Korea, such as the prevalence of mother’s status for HBV antigen and for the high viral load of HBV, in addition to the coverage and effectiveness of a particular intervention. The basic assumption of the model is that mother-to-child transmission is the only channel of HBV infection for the birth cohort, focusing on the preventive effect of intervention strategies on perinatal infection of HBV [17].

**Fig. 2:**
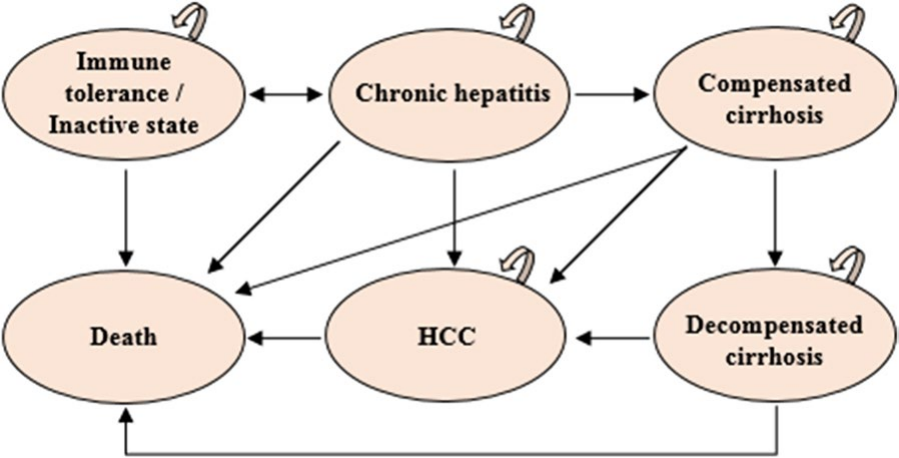
Markov diagram illustrating natural history of HBV. HBV, Hepatitis B Virus; HCC, hepatocellular carcinoma

This model also demonstrates the natural history of HBV infection among newborns of HBV-positive mothers via the Markov process (Fig. 2) [18]. In this process, there are eight distinct health states that indicate the progress of HBV morbidity from the susceptible state [17, 19–21] (Not infected/Recovery, Immune tolerance/Inactive state, Chronic hepatitis, Compensated cirrhosis, Decompensated cirrhosis, HCC, Disease-specific death, and All-cause death). The Markov process terminates either when all newborns reach their death state or age 100. Each Markov cycle is equivalent to 1 year. A half cycle correction was applied to estimate the costs and effectiveness [18]. The decision model was built using TreeAge Pro 2017 (TreeAge Software, Inc., Williamstown, MA).

### Epidemiological parameters and intervention coverages

We differentiated the prevalence of HBV infection among pregnant women into three parts, according to the presence of HBsAg, hepatitis B envelope antigen (HBeAg), and high viral load, because the risk of perinatal infection is respective to their status of antigen and viral load [22]. Due to the lack of national data for the proportion of high viral load among HBeAg positive mothers, we adopted this information from a Taiwanese study by Wen et al., which assessed the risk of perinatal HBV infection according to the levels of maternal viral load [23]. The risk of perinatal infection varied according to maternal HBeAg status, timing of birth dose, and HBIG administration [10, 24–31]. Moreover, the result from the meta-analysis examining the reducing effect of Lamivudine on the interruption of in utero transmission of HBV was chosen to represent the effect of antiviral prophylaxis [32]. This indicated that antiviral prophylaxis using Lamivudine may reduce the risk of perinatal infection by half.

The coverages of universal vaccination and current PHBPP, 90.7 and 99%, respectively, were considered in the model to reflect the real environment surrounding the vaccination programs in 2014 [10, 33]. Moreover, we assumed that the coverage of PHBPP with antiviral prophylaxis may increase to 100%, considering that a greater focus is paid to pregnant women enrolled in this program. To avoid the overestimation of benefits of the antiviral prophylaxis, we conducted a sensitivity analysis on its coverage by decreasing up to 50%. The parameters on the prevalence of HBV infection among pregnant women, risk of perinatal HBV infection, and intervention coverages are presented in Table 1 [10, 16, 23–34].

**Table 1:** Estimates of prevalence, effectiveness of vaccine & antiviral prophylaxis, and coverage

	Base-case value	Range	PSA distribution	PSA parameters	Source
Population and risk of perinatal infection (%)					
Total birth of 2014	435,435	–	–	–	[16]
Prevalence of HBsAg among pregnant women	2.45	1.7–3.2	Beta	α = 41.61; β = 1656.92	[7]
Prevalence of HBeAg among HBsAg(+) pregnant women	38.5	29.7–44.3	Beta	α = 64.37; β = 109.6	[10]
Prevalence of high viral load among HBeAg(+) pregnant women	80	60–100	Uniform	low = 0.6; high = 1	[23]
HBeAg(+) pregnant women					
HepB1 + HBIG within 24 h of birth	6.4	5.5–29	Beta	α = 6.96; β = 33.39	[10, 24–28, 30]
HepB1 within 24 h of birth	33.8	21–43	Beta	α = 22.7; β = 48.24	[26–28]
No HepB1 within 24 h of birth	87.5	62.6–96.7	Beta	α = 16.97; β = 4.34	[29]
HBeAg(−) pregnant women					
HepB1 + HBIG within 24 h of birth	1.4	0–3	Beta	α = 3.93; β = 257.74	[10, 25, 27, 30, 31]
HepB1 within 24 h of birth	6.6	0–13.2	Beta	α = 3.67; β = 51.94	[25, 27, 29]
No HepB1 within 24 h of birth	13.2	2.6–46.2	Beta	α = 3.54; β = 10.98	[29]
Reduction in perinatal infection with antiviral prophylaxis	50	37–85	Beta	α = 9.47; β = 6.05	[32]
Coverage of interventions (%)					
Antiviral prophylaxis	100	50–100	Beta	α = 8.25; β = 2.75	Assumed
HepB1 + HBIG within 24 h of birth	99	85–100	Beta	α = 44.71; β = 3.625	[10]
HepB1 within 24 h of birth	90.7	46.4–92.3	Beta	α = 10.5; β = 4.64	[33]

*HBsAg* hepatitis B Surface Antigen, *HBeAg* hepatitis B envelope antigen, *HepB1* hepatitis B birth dose, *HBIG* hepatitis B immune globulin, *24* *h* 24 hours, *PSA* probabilistic sensitivity analysis

Regarding the long-term progression of neonatal HBV infection, annual transition probabilities of health states in the Markov model were taken from the economic literature on vaccination strategies against HBV infection [17, 19, 20, 35, 36]. A clinical aspect of perinatal HBV infection—the high likelihood of developing to the immune tolerance phase among those infected with HBV during the perinatal period—was considered [37]. All-cause mortality was estimated using the life table of the cohort born in 2014 [38]. Specific values of the transition probabilities are listed in Table 2 [17, 19, 20, 35, 36].

**Table 2:** Transition probabilities for each cycle of the markov model (Unit: %)

	Base-case value	Range	PSA distribution	PSA parameters	Source
Perinatal infection to					
Immune tolerance/inactive state	89	80–90	Beta	α = 172.55; β = 30.45	[20, 35]
Immune tolerance/Inactive state to					
Chronic hepatitis (years)					
< 25	0.43	0.3–0.65	Beta	α = 29.32; β = 6144.29	[19]
≥ 25	3	2.9–7.3	Beta	α = 20.35; β = 378.64	[19]
Chronic hepatitis to					
Immune tolerance/inactive state (years)					
< 25	9	0–16.3	Beta	α = 3.59; β = 40.49	[19]
≥ 25	10	8.3–16.3	Beta	α = 33.05; β = 235.63	[19]
Compensated cirrhosis (years)					
< 25	0.065	0.01–0.12	Beta	α = 5.58; β = 8582.87	[19]
≥ 25	1.5	1–5.7	Beta	α = 7.82; β = 225.69	[19]
HCC	0.5	0.2–1	Beta	α = 8.94; β = 1481.06	[20, 36]
Disease-related death	0.9	0.3–3.6	Beta	α = 5.46; β = 274.46	[20, 36]
Compensated cirrhosis to					
Decompensated cirrhosis	5.4	2.8–15	Beta	α = 7.67; β = 78.49	[20, 36]
HCC	3.3	0.5–6.6	Beta	α = 5.19; β = 141.04	[20, 36]
Disease-related death	3.5	0–8	Beta	α = 3.8; β = 91.2	[17, 20]
Decompensated cirrhosis to					
HCC	7.1	0.15–10	Beta	α = 3.98; β = 74.46	[17, 20, 36]
Disease-related death	15	9.9–50	Dirichlet	List (20;75;5)	[20, 36]
HCC to					
Disease-related death	54	8.1–70	Dirichlet	List (20;75;5)	[20, 36]

*HCC* hepatocellular carcinoma, *PSA* probabilistic sensitivity analysis

### Costs estimations

Costs in this study were evaluated from a healthcare system perspective, including intervention program costs, direct medical costs, and direct non-medical costs [39]. All cost estimates were based on the year 2014, and the South Korean Won was converted to US dollars ($1 = ₩1053.3). Table 3 summarizes the detailed information.

**Table 3:** Estimates of cost parameters, utility, and discount rate

	Base-case value	Range	PSA distribution	PSA parameters	Source
Annual expenditure by the government (USD)					
Budget of PHBPP in 2014	1,634,281	–	–	–	[33]
HBV vaccine per capita	60	48–72	Uniform	low = 48; high = 72	[9]
Antiviral prophylaxis per capita	369	178–559	Uniform	low = 178; high = 559	[40]
HBV-DNA testing per capita	66	53–79	Uniform	low = 53; high = 57	[41]
Direct medical cost per capita (USD)					
Chronic hepatitis					
Inpatient	248	± 20%	Gamma	α = 0.0167; λ = 0.0001	–
Outpatient	415	± 20%	Gamma	α = 0.134; λ = 0.0032	–
Pharmaceuticals	157	± 20%	Gamma	α = 0.09; λ = 0.00058	–
Compensated cirrhosis					
Inpatient	1504	± 20%	Gamma	α = 0.034; λ = 0.00002	–
Outpatient	473	± 20%	Gamma	α = 0.11; λ = 0.00023	–
Pharmaceuticals	125	± 20%	Gamma	α = 0.119; λ = 0.00095	–
Decompensated cirrhosis					
Inpatient	2602	± 20%	Gamma	α = 0.236; λ = 0.00009	–
Outpatient	189	± 20%	Gamma	α = 0.336; λ = 0.00178	–
Pharmaceuticals	53	± 20%	Gamma	α = 0.106; λ = 0.00199	–
HCC					
Inpatient	5161	± 20%	Gamma	α = 0.232; λ = 0.00005	–
Outpatient	1108	± 20%	Gamma	α = 0.217; λ = 0.0002	–
Pharmaceuticals	230	± 20%	Gamma	α = 0.096; λ = 0.00042	–
Direct non-medical cost (USD)					
Transportation cost (inpatient)	25	± 20%	Uniform	low = 20; high = 30	[33, 39]
Transportation cost (outpatient)	5	± 20%	Uniform	low = 4; high = 6	[33, 39]
Average number of inpatient visit					
Chronic hepatitis	0.07	0–0.14	Gamma	α = 0.027; λ = 0.379	–
Compensated cirrhosis	0.29	0–0.58	Gamma	α = 0.076; λ = 0.263	–
Decompensated cirrhosis	0.54	0–1.08	Gamma	α = 0.298; λ = 0.551	–
HCC	1.53	0–3.06	Gamma	α = 0.366; λ = 0.239	–
Average number of outpatient visit					
Chronic hepatitis	4.19	0–8.38	Gamma	α = 0.705; λ = 0.168	–
Compensated cirrhosis	4.62	0–9.24	Gamma	α = 0.847; λ = 0.183	–
Decompensated cirrhosis	2.38	0–4.76	Gamma	α = 0.442; λ = 0.186	–
HCC	8.38	0–16.76	Gamma	α = 0.704; λ = 0.084	–
Daily cost of caregiving (USD)	59	± 20%	Uniform	low = 47; high = 71	[33, 39]
Average days of hospitalization					
Chronic hepatitis	0.61	0–1.22	Gamma	α = 0.01; λ = 0.0168	–
Compensated cirrhosis	3.81	0–7.62	Gamma	α = 0.034; λ = 0.009	–
Decompensated cirrhosis	6.33	0–12.66	Gamma	α = 0.112; λ = 0.018	–
HCC	15.63	0–31.26	Gamma	α = 0.2102; λ = 0.0134	–
Average hours spending on outpatient visit	2	1–3	Uniform	low = 1; high = 3	[33, 39]
Utility					
Immune tolerance/inactive state	1	–	–	–	
Chronic hepatitis	0.73	0.63–0.98	Beta	α = 15.7; β = 3.803	[44–47]
Compensated cirrhosis	0.65	0.35–0.95	Beta	α = 5.92; β = 3.19	[44–47]
Decompensated cirrhosis	0.46	0.25–0.75	Beta	α = 7.5; β = 7.5	[44–47]
HCC	0.46	0.16–0.75	Beta	α = 4.73; β = 5.67	[44–47]
Discount rate (%)	5	3–7	–	–	[48]

*HCC* hepatocellular carcinoma, *PHBPP* Perinatal Hepatitis B Prevention Program, *PSA* probabilistic sensitivity analysis

To estimate the cost of each intervention, a combined approach was applied. In calculating the program cost of universal vaccination, a micro-costing method that encompassed the coverage of vaccination and its cost was used. Conversely, the cost of PHBPP was computed with a total budget of the program in 2014 by using a gross-costing method [33]. For PHBPP with antiviral prophylaxis, both methods—the micro-costing and the gross-costing—were incorporated in a way that the per capita cost of antiviral therapy and HBV-DNA tests were added to the total budget of PHBPP [40, 41].

To obtain information of direct medical and non-medical costs for patients with HBV-sequelae in Korea, we explored National Health Insurance Claims Database (NHICD) [42]. NHICD is managed by the National Health Insurance Service (NHIS), and contains information of the entire Korean population regarding their healthcare costs and utilizations (inpatient, outpatient, and pharmaceutical) within its insurance benefits package. For this study, we acquired the insurance claims dataset, including information of the entire Korean population experiencing HBV-sequelae based on International Classification of Diseases 10th Revision (ICD-10) at either a primary or secondary diagnosis code. The ICD-10 codes utilized in this study and the operational definitions are summarized to Appendix 1. This analysis using NHICD was approved by the Institutional Review Board of Seoul National University Hospital.

Direct non-medical costs of this study are composed of travelling cost, care-giving cost, and time cost, all of which requires information on healthcare utilization, such as the number of inpatient and outpatient visits, as well as hospitalization [39]. Thus, we analyzed the pattern of healthcare utilization among individuals diagnosed with HBV-sequelae from NHICD (Table 3). The same operational definition used for the cost analysis was applied uniformly for this investigation. We used different travelling costs for hospital utilization, reflecting that the mode of transportation may differ between inpatient and outpatient visits [33, 39]. Additionally, the care-giving cost was the daily salary for a professional care-giver during hospitalization, which was obtained from the Korea Health Panel [39]. To calculate the time cost, we estimated the cost of labor loss during outpatient visits by using both the employment rate and average hourly payment [43].

### Health outcome measures

We evaluated the effectiveness of this study by QALYs, as well as new cases of HBV-related morbidity and the disease specific mortality. QALY estimates of HBV-sequelae were adopted from the study of utility weights for major liver diseases in Korea as well as several economic evaluation studies [44–47]. Following the recommendation of the Health Insurance Review & Assessment Service in Korea, both costs and QALYs were discounted at 5% [48]. The estimates of utilities and discount rate are presented in Table 3.

## Results

The results of cost-effectiveness analysis in this study are presented as incremental cost-effectiveness ratio (ICER), incidental cases of chronic hepatitis, compensated and decompensated cirrhosis, HCC, and HBV-related deaths. ICER is the difference in costs between the reference and the comparative strategy, divided by the difference in their QALYs. New cases of HBV-sequelae over the lifetime of the 2014 birth cohort are derived from the Markov cohort analysis, presenting the outcomes by per 100,000 people. The results are summarized in Tables 4 and 5.

**Table 4:** Costs, QALYs, and ICER of three intervention strategies

	Costs (USD)	Incremental costs	QALYs	Incremental QALYs	ICER
PHBPP (reference)	28,236,374	–	8,462,686	–	–
PHBPP with antiviral prophylaxis	29,561,401	1,325,027	8,462,768	82	16,159
Universal vaccination	29,312,011	1,075,637	8,461,656	− 1030	(Dominated)

*PHBPP* Perinatal Hepatitis B Prevention Program, *QALY* quality-adjusted life years, *ICER* incremental cost-effectiveness ratio

**Table 5:** New cases of HBV-related diseases and deaths per 100,000 people

	Chronic hepatitis	Compensated cirrhosis	Decompensated cirrhosis	HCC	HBV-related deaths
PHBPP (reference)	106	12	0	0	13
PHBPP with antiviral prophylaxis	72	8	0	0	0
Universal vaccination	567	56	22	37	97

*HCC* hepatocellular carcinoma

Considering that PHBPP, which is the current strategy for preventing perinatal HBV infection in Korea, was set as the reference, ICER of PHBPP with antiviral prophylaxis was $16,159. Universal vaccination, on the other hand, was dominated by the reference since the former strategy produced not only higher costs, but also less total QALYs than the latter strategy.

Regarding the new cases of HBV-sequelae per 100,000 people, PHBPP with antiviral prophylaxis averted 34 cases of chronic hepatitis, 4 cases of compensated cirrhosis, and 13 cases of HBV-related deaths per 100,000 people over the lifetime compared with the reference.

**Fig. 3:**
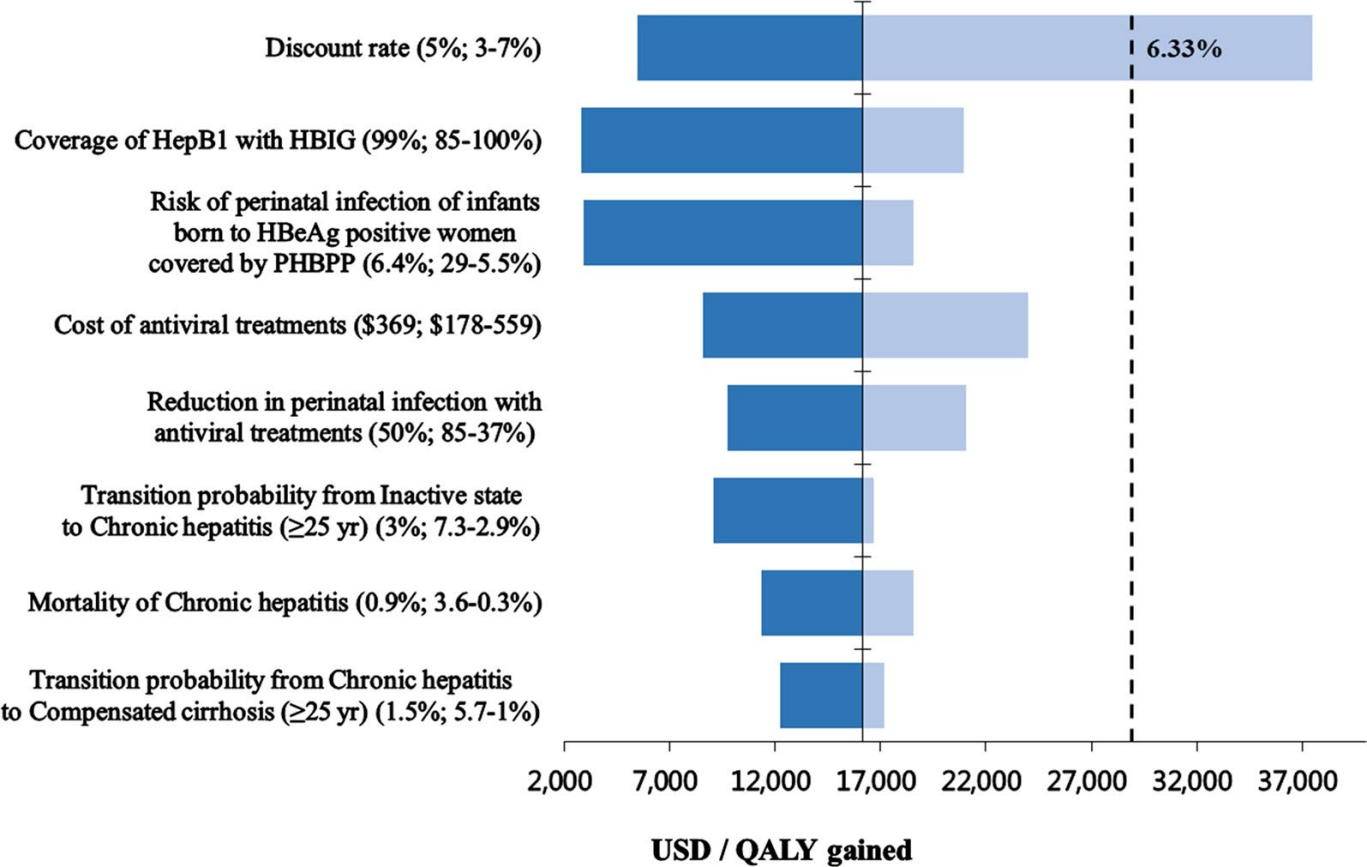
Tornado diagram presenting one-way sensitivity analyses (PHBPP with antiviral prophylaxis vs. current PHBPP). HBeAg, Hepatitis B Envelope Antigen; HepB1, Hepatitis B Birth Dose; HBIG, Hepatitis B Immune Globulin; QALY, quality-adjusted life years

To assess the robustness of the results, we conducted both deterministic and probabilistic sensitivity analyses. All parameters in this study were evaluated using a series of one-way sensitivity analyses, and the eight parameters showing a large variation of ICER were presented in the tornado diagram comparing PHBPP with antiviral prophylaxis to current PHBPP (Fig. 3). Moreover, we attempted to assess their threshold when setting the willingness-to-pay (WTP) to $29,000/QALY, a gross domestic product per capita in Korea during the year 2014 [43]. The results indicated that the discount rate of higher than 6.33% may affect robustness of the outcome. To further understand the impact of the discount rate on the ICER, we presented results under two different discounting scenarios (Appendix 2): (A) no discount rate was applied to both costs and QALYs, and (B) a lower discount rate was used for QALYs (2.5%) than for costs (5%). The results demonstrated that PHBPP with antiviral prophylaxis in Scenario A was cost-saving, and the ICER of this strategy in Scenario B was $4,926 compared to current PHBPP.

**Fig. 4:**
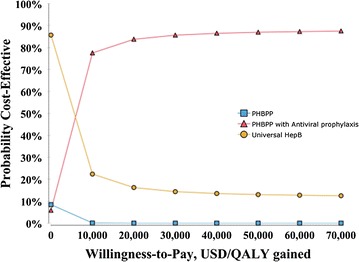
Cost-effectiveness acceptability curve. PHBPP, Perinatal Hepatitis B Prevention Program; HepB, Hepatitis B Vaccination; QALY, quality-adjusted life years

The result of probabilistic sensitivity analysis (i.e. Monte Carlo Simulation using 100,000 iterations) is presented as the cost-effectiveness acceptability curve (CEAC) in Fig. 4, indicating the probability of cost-effectiveness through a variation of WTP. The results showed that among the three preventive strategies against perinatal HBV infection, PHBPP with antiviral prophylaxis had approximately a 85% chance to be cost-effective when WTP for an additional QALY was over $20,000.

## Discussion

This study demonstrated that PHBPP with antiviral prophylaxis for women with a high viral load of HBV (≥ 10^6^ copies/mL) during pregnancy may increase the quality of life and prevent HBV-related deaths in newborns when compared with PHBPP without antiviral prophylaxis. Moreover, this study showed that the antiviral prophylaxis intervention may be cost-effective from a healthcare system perspective according to WTP in the Korean society. The result is sensitive to one factor, indicating that PHBPP with antiviral prophylaxis may not be cost-effective if the discount rate exceeds 6.33%. This well explains the effect of discounting on the cost-effectiveness of prevention programs where most costs occur early in the programs and most health benefits are realized in the future [49]. In this case, the ICER becomes less favorable as a higher discount rate is applied. Thus, to incorporate the value of future health benefits into decision making, adopting differential discounting could be considered as shown in Appendix 2 [50]. Our study also evaluated the cost-effectiveness of the current PHBPP compared with universal vaccination; the results showed that PHBPP may be superior with respect to cost-effectiveness.

If the provision of antiviral prophylaxis is incorporated, it appears that there could be a significant reduction in the economic and disease burden of HBV-related diseases in Korea. Since high mortality and morbidity of liver diseases are mostly associated with an economically active population, a large proportion–84.4%–of economic burden stemmed from premature death and absence from work; the total cost was estimated to be about $5401 million in 2008 [51]. Moreover, the country’s burden of liver diseases is not negligible in that cirrhosis and HCC are ranked 3rd and 10th among males, and 8th and 45th among females by disability-adjusted life years (DALYs) [52]. Given that HBV infection is the primary cause of liver diseases in Korea, an alternative strategy that supplies antiviral prophylaxis to women with HBV during pregnancy may promote health and productivity of the overall population in Korea by preventing perinatal HBV infection.

Currently, economic evaluations of antiviral prophylaxis as a means to prevent vertical transmission of HBV have been implemented in several countries, including the United States (U.S), Taiwan, and China [15, 53, 54]. These evaluations suggest that an incorporation of antiviral prophylaxis to the existing immunoprophylaxis strategy against perinatal HBV infection can be cost-effective, corresponding to our analysis. Although these studies showed a large variation in the prevalence of HBsAg in pregnant women (from 0.6% of the U.S to 9.5% of Taiwan), the robustness of outcomes was not susceptible to the epidemiological status of HBsAg in each society. This may improve the decision-making process regarding the introduction of antiviral therapy to women who are HBsAg-positive during their late pregnancy.

This study has several limitations to consider. Our model did not take into account the risk of adverse events following antiviral prophylaxis during pregnancy. However, numerous literature support that antiviral agents, such as Lamivudine, Telbivudine, and Tenofovir, hardly yield any medical complications, like birth defects [54]. Furthermore, it was challenging to estimate the direct medical costs per capita using NHICD, considering that a high proportion of medical services for liver diseases is not entirely covered by NHIS [55]. To address this, we applied the proportion of insurance coverage of HBV-sequelae, which Yang et al. obtained by investigating the medical records of patients with HBV-sequelae in 2006 from four tertiary hospitals in Korea (Appendix 3), to acquire the total amount of direct medical costs in 2006 consisting of NHIS payment and out-of-pocket payment by an individual patient with HBV-sequelae [55]. The costs of 2006 were inflated to the value of 2014 with an application of price index of inpatient, outpatient, and pharmaceuticals [56, 57]. Moreover, because some parameters in the model, such as transition probabilities of HBV-sequelae, were adopted from economic evaluation studies performed in other countries, the result of our study may not be perfectly applicable to the Korean health system. By implementing sensitivity analysis, however, we tried to advocate robustness of the results in this study. Lastly, since the time horizon of our model was the entire lifespan of the 2014 birth cohort, the projected outcomes reported as a net present value in this study may not support decision making for budget planning. This is because budgeting requires precise estimates of costs and outcomes in accordance with relevant time horizons of budget holders which is normally 1–5 years [58]. An additional budget impact analysis of introducing antiviral prophylaxis during pregnancy needs to be implemented to illustrate financial flows for each budget period after the onset of the intervention.

## Conclusions

This study implies that in Korea, PHBPP with antiviral prophylaxis may be the most cost-effective strategy under WTP of $29,000/QALY, and that this strategy is likely effective in reducing the burden of liver diseases by preventing mother-to-child transmission of HBV. Therefore, it is advisable to augment the current PHBPP in Korea by supplying antiviral therapy to women with a high viral load of HBV (≥ 10^6^ copies/mL) during their late pregnancy.

## Appendix 1. Operational definitions of HBV-related diseases

**Table:**

Disease category	Operational definition (ICD-10)
Chronic hepatitis	Contains B18 at a primary or secondary diagnosis code, and does not contain K74, C22 at a primary or secondary diagnosis code
Compensated cirrhosis	Contains K74 at a primary or secondary diagnosis code, and does not contain I85, K65, R18, R60, R17, K76.6, K76.7 at a primary or secondary diagnosis code
Decompensated cirrhosis	Contains K74 at a primary or secondary diagnosis code, and contains I85, K65, R18, R60, R17, K76.6, K76.7 at a primary or secondary diagnosis code
Hepatocellular carcinoma	Contains C22 at a primary or secondary diagnosis code, and contains V027, V193, V194 at a cancer confirmation code

## Appendix 2. Results of cost-effectiveness analysis under different discounting scenarios

**Table:**

	Costs	Incremental	QALYs	Incremental	ICER
	(USD)	Costs		QALYs	
*Scenario A—undiscounted*					
PHBPP (Reference)	32,336,200		35,613,756		(Dominated)
PHBPP with antiviral prophylaxis	32,295,959	− 40,241	35,614,910	1154	Cost saving
Universal Vaccination	54,929,313	22,593,113	35,599,124	− 14,632	(Dominated)
*Scenario B—costs (5%) and QALYs (2.5%)*					
PHBPP (reference)	28,236,374	–	14,918,008		
PHBPP with antiviral prophylaxis	29,561,401	1,325,027	14,918,277	269	4926
Universal vaccination	29,312,011	1,075,637	14,914,601	− 3407	(Dominated)

## Appendix 3. Proportion of insurance benefit coverage for HBV-related diseases

**Table:**

	Inpatient	Outpatient	Pharmaceuticals
Chronic hepatitis	0.35	0.50	0.46
Liver cirrhosis	0.45	0.74	0.57
Hepatocellular carcinoma	0.59	0.81	0.31
